# Supplementary material for: Glycemic control and bacterial infectious risk in type 2 diabetes: A retrospective cohort from a primary care database
Source: PLoS One. 2024 Dec 13;19(12):e0314287. doi: 10.1371/journal.pone.0314287 (PMC11642988; doi:10.1371/journal.pone.0314287)
Supplement: Attachment — Submitted filename: Responses to Reviewers.docx [file pone.0314287.s001.docx]

Rebutal Letter, Plos One

dear reviewers,

First of all, thank you for taking the time to read our article and for your feedback. You have both pointed out a lack of clarity regarding patient inclusion and exclusion criteria. A detailed flow chart is now available as an attachment to the article.

I'll answer your questions and comments one by one.

**Journal requirements :**

**When submitting your revision, we need you to address these additional requirements. Please ensure that your manuscript meets PLOS ONE's style requirements, including those for file naming. The PLOS ONE style templates can be found at 
https://journals.plos.org/plosone/s/file?id=wjVg/PLOSOne_formatting_sample_main_body.pdf and** 
[**https://journals.plos.org/plosone/s/file?id=ba62/PLOSOne_formatting_sample_title_authors_affiliations.pdf**](https://journals.plos.org/plosone/s/file?id=ba62/PLOSOne_formatting_sample_title_authors_affiliations.pdf)**.**

The title page has been updated to meet PLOS ONE’s style requirements.

**Please provide additional details regarding participant consent. In the ethics statement in the Methods and online submission information, please ensure that you have specified (1) whether consent was informed and (2) what type you obtained (for instance, written or verbal, and if verbal, how it was documented and witnessed). If your study included minors, state whether you obtained consent from parents or guardians. If the need for consent was waived by the ethics committee, please include this information.**

**If you are reporting a retrospective study of medical records or archived samples, please ensure that you have discussed whether all data were fully anonymized before you accessed them and/or whether the IRB or ethics committee waived the requirement for informed consent. If patients provided informed written consent to have data from their medical records used in research, please include this information**

Data were fully anonymized, and we had no access to identifying data before, during and after the study. Under French regulations, retrospective database studies do not require patient consent. They do, however, require information, which has been provided by posters in physicians' offices as recommended by the CNIL Commission nationale de l'informatique et des libertés (French Data Protection Authority). If patients did not wish their data to be used, they could contact the PRIMEGE Normandie data manager to have their data withheld.

We uploaded the Rules of Procedure of the scientific and ethical committee of the PRIMEGE health database.

**Note from Emily Chenette, Editor in Chief of PLOS ONE, and Iain Hrynaszkiewicz, Director of Open Research Solutions at PLOS: Did you know that depositing data in a repository is associated with up to a 25% citation advantage (https://doi.org/10.1371/journal.pone.0230416)? If you’ve not already done so, consider depositing your raw data in a repository to ensure your work is read, appreciated and cited by the largest possible audience. You’ll also earn an Accessible Data icon on your published paper if you deposit your data in any participating repository (**[**https://plos.org/open-science/open-data/#accessible-data**](https://plos.org/open-science/open-data/#accessible-data)**).**

**We note that you have indicated that there are restrictions to data sharing for this study. PLOS only allows data to be available upon request if there are legal or ethical restrictions on sharing data publicly. For more information on unacceptable data access restrictions, please see http://journals.plos.org/plosone/s/data-availability#loc-unacceptable-data-access-restrictions.**

**Before we proceed with your manuscript, please address the following prompts:**

**a) If there are ethical or legal restrictions on sharing a de-identified data set, please explain them in detail (e.g., data contain potentially identifying or sensitive patient information, data are owned by a third-party organization, etc.) and who has imposed them (e.g., a Research Ethics Committee or Institutional Review Board, etc.). Please also provide contact information for a data access committee, ethics committee, or other institutional body to which data requests may be sent.**

**We will update your Data Availability statement on your behalf to reflect the information you provide**

Access to aggregated raw data is possible through a request to PRIMEGE Normandie's ethical and scientific committee as described in the database's internal regulations, either by contacting the data manager at primege.normandie@univ-rouen.fr, or by contacting the doctor in charge of IT at [mikaeldusenne@gmail.com](mailto:mikaeldusenne@gmail.com).

Other researchers have the option of redoing the analyses, reproducing the studies carried out following a request to the ethical and scientific committee. The application form is available in the rules of procedure of the Ethics and Scientific Committee.

**Reviewer 1 :**

Q1 : **Were sample size calculation carried out? How was it done? This needs to be detailed in the manuscript**

The number of subjects required was based on the incidence rates of bacterial infections in T2DM patients with an HbA1c of between 5.5 and 6.5% and those with an HbA1c of between 8.5 and 9.5% in the Mor et al study (1), for an alpha risk of 5% and a beta risk of 20%, giving a required number of subjects of 276.

1. Mor A, Dekkers OM, Nielsen JS, Beck-Nielsen H, Sørensen HT, Thomsen RW. Impact of Glycemic Control on Risk of Infections in Patients with Type 2 Diabetes: A Population-Based Cohort Study. American Journal of Epidemiology. 2017;186(2):227‑36

Q2 : **There is no consensus on the impact of glycemic control on this infectious risk and the results of studies on this subject seem heterogeneous, lack of information regarding the heterogeneous results**

Concerning the heterogeneity of results from other studies, results from studies investigating the link between glycemic control and incidence of infection are heterogeneous, with a significant difference depending on whether the study population is from primary care or hospital recruitment, and on the methods or the definition used to determine glycemic control or infectious risk. The study by Pearson et al. (1) identified a need for research into the impact of long-term glycemic control, particularly HbA1c, and infectious susceptibility in diabetic patients.

We may need to clarify in the sentence you quote "impact of long-term glycemic control" which is justified by the literature review by Pearson et al and detail the criteria by which we judge these studies to be heterogeneous.

1. Pearson-Stuttard J, Blundell S, Harris T, Cook DG, Critchley J. Diabetes and infection: assessing the association with glycaemic control in population-based studies. The Lancet Diabetes & Endocrinology. 1 févr 2016;4(2):148‑58

**Q3 : What’s the follow-up criteria of overall population, as “The patients included had to be aged 18 years and older, had type 2 diabetes, and at least three visits in a primary care center” is for population with type 2 diabetes.**

- The follow-up criteria of the overall population are the same as for type 2 diabetes patients, i.e. be aged 18 years and older and at least 3 consultations at the primary care center.

**Q4 : A flow chart of study identification, inclusion and exclusion criteria will be helpful.**

- The flow chart is now done. (Fig 1)

**Reviewer 2 :**

**Q1 : Although the author provided detailed explanations on the inclusion criteria, the exclusion criteria were not clear enough. For example, diabetes patients with other diseases may also increase the risk of infection.**

You are perfectly right to point out that other chronic diseases are likely to increase the risk of infection (COPD, HIV, heart failure, etc.), as shown by the example of invasive pneumococcal infections (1). We did not adjust for other chronic diseases increasing the risk of infection, and their prevalences were probably significant in the different groups. This is a limitation that we must indeed stipulate in the article and should be taken in consideration in further studies.

The retrospective cohort study design makes it possible to exclude as few patients as possible, with the aim of increasing the statistical power of the study and carrying out a real-life study.

1. Moe H. Kyaw, Charles E. Rose, Alicia M. Fry, James A. Singleton, Zack Moore, Elizabeth R. Zell, Cynthia G. Whitney, for the Active Bacterial Core Surveillance Program of the Emerging Infections Program Network, The Influence of Chronic Illnesses on the Incidence of Invasive Pneumococcal Disease in Adults, The Journal of Infectious Diseases, Volume 192, Issue 3, 1 August 2005, Pages 377–386, <https://doi.org/10.1086/431521>

**Q2 : The statistical analysis in this article is not very appropriate. In table 1, the overall population should include the population with type2 diabetes, so the description of the antidiabetic treatments is incorrect. It is better to compare the diabetics who had an infection with diabetic case-controls who did not have an infection to ensure consistency between the baselines of the two groups or whether the conclusions are influenced by other factors.**

- For statistical analysis, the ANOVA test can be used to determine whether at least one of the groups has a statistically different result from the others, which is not the case in our study. Statistical analysis of multiple groups is made difficult here by the heterogeneity of the size of the different groups.
- Concerning table 1, We have modified the table by adding a 3rd column including non-diabetic patients for greater clarity and corrected the Overall Population column. However, we did not search for anti-diabetic treatments in non-diabetic patients, so the proportions of the number of treatments are probably wrong in this population. Perhaps we should therefore leave the term NA for these results.
- On your recommendation, we carried out a case-control study of diabetic patients who had received at least one antibiotic prescription, compared with those who had not. The results enabled us to identify certain potentially confounding factors, such as gender and BMI, although further statistical analysis showed their impact to be low.

In addition, glycated hemoglobin was not different between the two groups, supporting the main findings of the study. We add a table with results. (Table 3)

**Q3 : Similarly, in Table 2, we still do not know whether the two groups are matched. And whether the mean of antibiotic prescription per year has undergone a logarithmic transformation? Moreover, the “SD value” is greater than the “mean value”, making the data confusing.**

- The population studied is composed solely of identified type 2 diabetics, who have been separated and analyzed into several groups according to their glycemic control, the number of antidiabetic treatments and the type of antidiabetic treatment, without any matching between the different groups.
- Concerning the logarithmic transformation, it only concerns the graphical representation in boxplot for reasons of visual clarity.
- Regarding standard deviations that are higher than the mean, this can be explained by the fact that most values are quite low with a long tail of high values (skew). This can be seen with values that can only be positive, with a concentration towards zero, and whose distribution follows a multiplicative mode.

**Q4 : Some references are in French and English should be used uniformly.**

References have been translated.

Thank you again for your interest in our study and for helping to improve it. You have our gratitude.

If you have any further questions or comments, please do not hesitate to contact us.

Dr Lemoine Edouard
