# Supplementary material for: Glycemic control and bacterial infectious risk in type 2 diabetes: A retrospective cohort from a primary care database
Source: PLoS One. 2024 Dec 13;19(12):e0314287. doi: 10.1371/journal.pone.0314287 (PMC11642988; doi:10.1371/journal.pone.0314287)
Supplement: Attachment — Submitted filename: Rebutal Letter 2 (1).odt [file pone.0314287.s002.odt]

Rebutal Letter, Plos One
Dear reviewers, 

Thank you again for your interest in our article and for your comments and suggestions, which have greatly improved its quality. 

Reviewer 1:

1 Please check the spelling of HbAlc in Table 3.

We have harmonized all HbA1c abbreviations.

2.Please make sure that the ways of the representation of continuous variables （mean（SD）or mean ± SD） are consistent in Tables 1, 2, and 3.

All standard deviations have been harmonized in the tables.

Reviewer 2: 

Despite revising most of the review comments, the authors did not address two key issues. Firstly, the potential impact of other major diseases was not ruled out, especially considering the possibility that widespread neocoronary epidemics could elevate the risk of bacterial infections. Secondly, there was a lack of baseline comparison between diabetic patients without infections and those with infections. Furthermore, age and duration of diabetes are significant factors that may affect bacterial infection, yet no corrective analysis was conducted to account for these variables.

To improve identification of confounding factors, we supplemented the case-control section with other variables: Vaccination rate, smoking status, number of chronic treatments on the usual prescription, number of antidiabetic treatments, history of malignancy, COPD/Asthma or Cardiovascular event. The results have been presented in table 4.
We also performed a multivariate analysis using a negative binomial model, taking into consideration BMI, age, smoking status, HbA1c threshold, history of asthma and COPD, cardiovascular event and history of malignancy. This required us to design new pathology detection algorithms in the database.
This analysis enabled us to confirm our results and to identify confounding factors and the strength of their impact on the number of antibiotic prescriptions. The results are presented in Table 3. 
Concerning the duration of diabetes, this is an important factor which is difficult to estimate in our database, as it is poorly structured. Nevertheless, this factor could be studied in the future, once the database has been linked with health insurance data.

Thank you for your comments, we hope we've answered them well.

Dr Lemoine Edouard, MD
